# Translation of Cellular Senescence to Novel Therapeutics: Insights From Alternative Tools and Models

**DOI:** 10.3389/fragi.2022.828058

**Published:** 2022-06-01

**Authors:** Nurcan Inci, Dilanur Kamali, Erdogan Oguzhan Akyildiz, Eda Tahir Turanli, Perinur Bozaykut

**Affiliations:** ^1^ Graduate School of Natural and Applied Sciences, Acibadem Mehmet Ali Aydinlar University, Istanbul, Turkey; ^2^ Department of Molecular Biology and Genetics, Faculty of Engineering and Natural Sciences, Acibadem Mehmet Ali Aydinlar University, Istanbul, Turkey

**Keywords:** Cellular senescence, Aging, Senotherapeutics, Naked mole-rat, Blind mole-rat, Cardiovascular Diseases, Organoid, Microchip

## Abstract

Increasing chronological age is the greatest risk factor for human diseases. Cellular senescence (CS), which is characterized by permanent cell-cycle arrest, has recently emerged as a fundamental mechanism in developing aging-related pathologies. During the aging process, senescent cell accumulation results in senescence-associated secretory phenotype (SASP) which plays an essential role in tissue dysfunction. Although discovered very recently, senotherapeutic drugs have been already involved in clinical studies. This review gives a summary of the molecular mechanisms of CS and its role particularly in the development of cardiovascular diseases (CVD) as the leading cause of death. In addition, it addresses alternative research tools including the nonhuman and human models as well as computational techniques for the discovery of novel therapies. Finally, senotherapeutic approaches that are mainly classified as senolytics and senomorphics are discussed.

## Introduction

Cellular Senescence (CS), defined as irreversible cell-cycle arrest, has become popular in recent years due to its high association with aging and age-related diseases. Although CS is a defense mechanism against damage or stress factors, its accumulation during aging has been proposed to cause many age-related pathologies. CS was first discovered in human diploid cells (fibroblast cells) in the early 1960s by Hayflick and Moorhead. They observed that fibroblasts reach the maximum number of cell divisions before they go through irreversible cell cycle arrest (Hayflick and Moorhead, 1961) which was later named replicative senescence (RS). Although, the cause of CS was stated as telomere shortening at first (Harley et al., 1990), and that telomerase bypasses the senescence arrest (Bodnar et al., 1998), senescence may also be triggered by other aging-associated stimuli (López-Otín et al., 2013).

CS, which is characterized by irreversible cell cycle arrest as a response to different stressors, is considered as a double-edged sword depending on the cellular and physiological processes. CS may both have beneficial and harmful effects representing evolutionary antagonistic pleiotropy (Giaimo and d’Adda di Fagagna, 2012) while CS has beneficial effects on organisms during various pathological and physiological processes such as wound healing and tumor suppression, it also has harmful effects on organisms, especially, during the aging process (He and Sharpless, 2017). The irreversible growth-arrested cells turn into senescent cells that secrete proinflammatory cytokines, growth factors, and proteases named senescence-associated secretory phenotype (SASP) through autocrine/paracrine pathways (Kumari and Jat., 2021). In paracrine fashion, secretion of large amounts of SASP harms the neighboring cells, and eventually, causes them to become senescent cells and increases the SASP secretion (Bozaykut, 2019).

Although CS mechanisms involve complex mechanisms (Karin and Alon, 2021), recent work suggests removing CS aids to prevent age-related pathologies. During aging, various scenarios are possible. CS accumulation may either result from increasing production of senescent cells with age or arise from decreasing removal of senescent cells, again with age (Florido et al., 2011; Kirkland and Tchkonia, 2017; Karin and Alon, 2021). Overall, CS is highly associated with aging which is a progressive process characterized by the functional decline and dysfunction in cells and tissues (McHugh and Gil, 2017; Rodrigues et al., 2021) that ultimately causes various age-related diseases, including cardiovascular disease (CVD) (Parry and Narita, 2016; Campisi, 2018; Bozaykut, 2019). Recently, to prevent tissue dysfunction and age-related pathologies, removal of senescent cells or ameliorating CS phenotype by senotherapeutic drugs has been proposed as a promising therapeutic approach. Moreover, using omics technologies and bioinformatic approaches, repurposing of the drugs could be a promising and rapid approach for the discovery of new senotherapeutics.

In this review, the current field of CS will be surveyed with a special emphasis on CVD by summarizing its essential role and important bottlenecks in the area. In addition, the utilization of alternative aging models such as long-lived and short-lived rodents and computational approaches in order to identify the molecular mechanisms of CS and to develop novel senotheraupetics will be discussed.

## Common Pathways of Cellular Senescence

**FIGURE 1:**
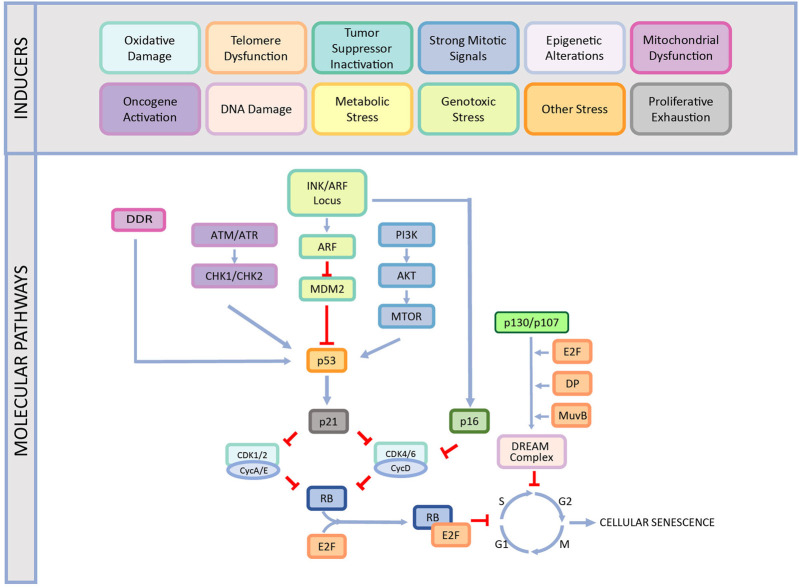
The causes and different mechanisms involved in cellular senescence. CS is induced by various factors such as oxidative damage, telomere dysfunction, tumor suppressor inactivation, strong mitogenic signals, epigenetic alterations, mitochondrial dysfunction, oncogene activation, DNA damage, proliferative exhaustion, metabolic stress, genotoxic stress, and other stress. This induction occurs by different pathways and ends up with stable cell cycle arrest. This figure is created by BioRender.com.

While CS is an essential cellular response to different stress factors or developmental signals, it is also one of the primary causes of aging and aging-related diseases (López-Otín et al., 2013; Surova and Zhivotovsky, 2013; Galluzzi et al., 2018; Sapieha and Mallette, 2018; Bozaykut et al., 2020b). Therefore, understanding the underlying mechanisms of CS is crucial to offer therapeutic approaches for these diseases. There are various molecular mechanisms that play a role in the development of the CS including DNA damage response, cell cycle arrest, p53/p21^WAF1/CIP1^ pathway, INK/ARF locus, p16^INK4A^/pRB pathway as reviewed below in detail and summarized in Figure 1.

### DNA Damage Response

DDR, which is an evolutionarily conserved response to DNA damage, gets activated by single-strand or double-strand breaks of DNA. When the cell comes across irreparable DNA damage, two possible cellular fates are either apoptosis or CS to prevent the proliferation of the damaged cells. In this regard, DDR machinery determines the cell fate based on the extent and duration of the DNA damage signaling. While short-term DNA damage mainly leads to apoptosis, long-term mild DNA damage leads to CS (Petrova et al., 2016).

During the replicative senescence process, the progressive telomere shortening in human diploid cells causes unprotected, double-stranded chromosomal free ends and these uncapped ends are identified by DDR machinery (d’Adda di Fagagna et al., 2003; Shay and Wright, 2019). On the other hand, intrinsic stimuli such as hyperproliferation, telomere attrition, oxidative damage, and extrinsic stimuli such as chemotherapeutic drugs, γ-irradiation, ultraviolet lead to persistent DDR signaling to result in irreversible and irreparable DNA damage and eventually in CS (Di Leonardo et al., 1994; D’Adda Di Fagagna, 2008; Fumagalli et al., 2012).

Yet, another stressor is oncogene activation named oncogene-induced senescence (OIS) that causes a hyperproliferative phase. During the OIS, mitotic signals trigger DNA replication and cause genomic damage accumulation and consequently, DDR gets induced. Although OIS is mediated by both DDR and tumor suppressor mechanisms, DDR is more sensitive and requires a lower oncogenic load (Evangelou and Ioannidis, 2013; Gorgoulis et al., 2018). While in RS, DDR depends on the telomeric length. In OIS, DDR does not depend on telomeric length even though it is associated with telomeric dysfunction (d’Adda di Fagagna et al., 2003; Suram et al., 2012). However, it has been proven that both telomeric and non-telomeric DNA damage have equivalent roles in triggering senescence (Nakamura et al., 2008). Based on this information, the relationship between DNA damage and CS is undeniably strong. Consistently, increased oxygen concentration just after birth leads to DNA-damage response which is followed by cell cycle arrest in the cardiomyoctes (Puente et al., 2014) and the rate of cardiomyocytes turnover has also been shown to decline with aging (Bergmann et al., 2015). Moreover, recently, it was suggested that in the failing heart which is accompanied by DNA damage, deprivation of α-myosin heavy chain (α-MHC) and elevation of β-MHC leads to inadequate response to traditional therapies (Katsuumi et al., 2018). Another study performed in aging mice showed that the loss of α-MHC gene through DNA damage induces cardiomyocyte apoptosis, heart failure, cardiac contractile dysfunction, and left ventricular hypertrophy (Barouch et al., 2006) pointing out the fact that DNA damage affects both functionality of the cardiac cells and drug responses in the older people although underlying mechanisms are still not clearly defined (Jackson et al., 2021).

### Cell Cycle Arrest

The cell cycle is highly essential for cellular processes such as development, proliferation, and viability and one of the main characteristics of senescent cells is stable cell cycle arrest. When the dysfunctional cells go down the stable cell cycle arrest which is a defense mechanism triggered by different stressors, the proliferation of dysfunctional cells is halted and the cell cannot continue to divide. Cell cycle arrest occurs in G1 and G2 phases in senescent cells (Mao et al., 2012; Gire and Dulic 2015; Kumari and Jat, 2021) and the G0 phase in the quiescent cells (Di Leonardo et al., 1994; Kumari and Jat, 2021). Therefore, unlike quiescent cells, senescent cells cannot reenter the cell cycle even in favorable growth conditions because they cannot respond to mitogenic or growth factor stimulation while quiescent cells can proliferate when conditions are favorable (Campisi and d’Adda di Fagagna, 2007; Calcinotto et al., 2019; Gorgoulis et al., 2019; Mohamad Kamal et al., 2020). In addition, when considering various G0 states (G0-entry, G0 and G0-alert), it is important to distinguish them to understand the fate of cells and it is claimed that it can be identified with the molecular algorithm by checking Ki67, beta-galactosidase, and pRPS6 activities (Alessio et al., 2021).

During cell cycle arrest, nutrition sensory pathways, such as mTOR, stay active in growth arrest (Blagosklonny, 2013) implying that senescent cells are metabolically active even when the cycle is stopped. On the other hand, senescent cells are not apoptotic. Thus, they are not eliminated immediately. It is suggested that, under the stable activity of mTOR, the cells undergo stable growth arrest and become senescent, but when the mTOR activity is inhibited, senescence converted into a quiescent state retaining proliferative potential (Korotchkina et al., 2010; Blagosklonny, 2012). Therefore, the activity of the mTOR pathway is proposed to be an important factor in distinguishing one cell from another.

In addition, senescent cells are different from terminally differentiated cells that enter the irreversible cell cycle arrest. Terminal differentiation is a defined developmental program, whereas senescence is mostly executed as a cellular stress response (Baumann, 2016; Hinze and Boucrot, 2018; Mohamad Kamal et al., 2020). However, senescence may occur in terminally differentiated cells demonstrating that senescence is not dependent on an active cell cycle (Jurk et al., 2012; von Zglinicki et al., 2021). The activation of the p53/p21^WAF1/CIP1^ and p16^INK4A^/RB tumor suppressor pathways controls the cell cycle exit (Herranz and Gil, 2018; Kobashigawa et al., 2019; Liu and Wan, 2019) as discussed below.

### p53/p21^WAF1/CIP1^ Pathway

One of the common pathways that play a crucial role in senescent growth arrest is the p53/p21^WAF1/CIP1^ that is activated as a response to DNA damage caused by oxidative stress, oncogenic stress, and telomere attrition. After DNA damage occurs, p53 and, hence, p53 tumor suppressor pathway is activated by DDR signaling to regulate a set of transcription factors and antiproliferative genes (Olivier et al., 2010; Kastenhuber and Lowe, 2017). As a result, cells undergo irreversible growth arrest and become senescent (Lowe et al., 2004).

Since p53 has many roles within a cell, its regulation is driven by different factors. One of the most important functions of p53 is the induction of p21^CIP1^ transcription during CS progression. p21^CIP1^ is a cyclin-dependent kinase inhibitor (CDKi) and its inhibitory effects end up with hypophosphorylation of retinoblastoma protein (pRB) and formation of DREAM complex that is formed of p107 and p130 RB pocket proteins, resulting in the cell cycle exit (D’Adda Di Fagagna, 2008; Gire and Dulic, 2015). In addition to this, p53 signaling inactivation intervenes the initiation of CS (Shay et al., 1991; Beauséjour et al., 2003; Stewart et al., 2003; Campisi, 2005). According to several studies, CS may be disrupted by the inactivation of p53 (Shay et al., 1991; Beauséjour et al., 2003). The critical point in p53 induction is that; when the stress-induced senescence is temporary, it can induce the quiescence, and the DNA repair process becomes active to turn cells back to their usual cycle (Childs et al., 2015). However, chronic stress may cause long-term cell cycle arrest by activating p16^INK4A^, which is a CDK inhibitor (CDK4 and CDK6) (Sharpless and Depinho, 2006). Although, p21^CIP1^ induction is crucial for the initiation of senescence, its permanent expression is not required for senescent cells wherever p16^INK4A^ is maintained (Sharpless and Sherr, 2015). On the other hand, unlike p16^INK4A^ and p53, the upregulation of p21^CIP1^ is considered as the driver for developmental senescence (Muñoz-Espín et al., 2013; Storer et al., 2013).

The role of p53 has been also demonstrated in cardiac diseases through various studies. As such, p53 expression is observed to be highly expressed in the heart during cardiomyopathy when compared to the healthy heart. Furthermore, another study on murine models with overloaded left ventricular pressure showed that accumulation of p53 in cardiac cells leads to cardiac angiogenesis and impaired systolic function (Sano et al., 2007).

### p16^INK4A^/pRB Pathway

p16^INK4A^/pRB pathway is another important pathway during CS due to its significant impact on the cell cycle. As mentioned above, pRB is one of the tumor suppressor proteins that inhibit cell cycle progression from preventing excessive cell growth until the cell becomes ready for division. On the other hand, when pRB is phosphorylated, the cell cycle can maintain its progression. If p16^INK4A^ permanently activates pRB in human cells, the cells undergo irreversible cell cycle arrest and become senescent. Even if pRB is inactivated, the process is no longer revoked or reversed (Muñoz-Espín et al., 2013), which suggests that p16^INK4a^/RB pathway may induce an alternative way for the irreversible cell cycle arrest and collaborate with mitogenic signals to generate reactive oxygen species (ROS) that results in irreversible cytokinetic block (Takahashi et al., 2007).

The pRB belongs to the protein pocket family which can bind the functional region of other proteins by their specific pocket (Münger and Howley, 2002; Korenjak and Brehm, 2005). The main feature of pRB is its restriction ability in DNA replication to prevent the progression of the G1 phase to the S phase (Goodrich et al., 1991). In this regard, the dephosphorylated pRB binds and inactivates the E2F complexes by forming the RB-E2F complex. RB-E2F complex has a repressive capacity that eventually avoids the transcription of genes essential for the progress of the cell cycle (Wu et al., 1995; Funk et al., 1997; Fischer and Müller, 2017). Furthermore, to enhance the suppression of DNA synthesis, this repressive complex attracts factors such as histone deacetylases (HDACs) and histone methyltransferase SUV39H1 to inhibit transcription of S phase genes (Lai et al., 1999; Nielsen et al., 2001; Vandel et al., 2001). On the other hand, the hyperphosphorylation of pRB is linked to the inhibition at the restriction point. E2F cuts loose from the repressive complex, promoting the transcription of the S phase genes and the cell cycle progression (Zhang et al., 2000). In addition, the crosstalk between the pRB and AKT signaling pathways plays an important role in the switch of quiescence and senescence by re-coordinating the overlapping functions of Forkhead transcription factors such as FOXO3a and FOXM1 (Eijkelenboom and Burgering, 2013; Lam et al., 2013; Imai et al., 2014). Other than the role of RB-E2F complex in early S phase, DREAM complex, which consists of p107 and p130 with dimerization partner, E2F4-5 and a Multivulval class B (MuvB) is another master regulator of the cell cycle, mainly, of late S-phase and in G2 phase (Litovchick et al., 2007; Sadasivam et al., 2012). In addition to E2F binding sites, DREAM complex binds to cell cycle genes homology region promoter elements. Thus, it is suggested that it has larger effects and functions than RB for the control of the cell cycle (Müller et al., 2012; Guiley et al., 2015; Fischer and Müller, 2017). As such, DREAM dissociates with E2F/pRB components to bind B-MYB and FOXM1 activators to regulate the genes related to the cell cycle (Engeland, 2017).

The prominent role of the p16 pathway has been demonstrated in cardiovascular aging through several studies. As such, elevated p16 levels were demonstrated in the coronary arteries of hypertensive rat models (Westhoff et al., 2008; Boe et al., 2013). Another important study showed that p16^INK4a^ driven CS leads to alterations in the proinflammatory status of macrophages, therefore, affecting SASP levels (Cudejko et al., 2011). Finally, when p16-positive senescent cells were eliminated in premature aging mice, aging phenotypes were reduced in the heart, along with other tissues, resulting in the extension of healthspan (Baker et al., 2016).

### INK4/ARF Locus

INK4 family proteins have a crucial role in DNA repair, apoptosis, and CS (Cánepa et al., 2007). INK4 (short for INhibitors of CDK4) is a cyclin-dependent kinase inhibitors (CKIs) family that consists of p16^INK4A^ p15^INK4B,^ p18^INK4C^, and p19^INK4D^. These four members of the INK4 family are inhibitors of CDK4 and CDK6 (Li et al., 2015) which may lead to the blocking of cell cycle progression and thus, the cell cannot go past the G1 restriction point (Ortega et al., 2002).

On the other hand, ARF tumor suppressor proteins are encoded by the INK4/ARF locus while physically linking to each other. ARF regulates p53 stability by binding and inhibiting MDM2 (Sharpless, 2005; Kim and Sharpless, 2006), leading to a cross-talk between p53 and pRB pathways (Gil and Peters, 2006). On the other hand, p53 regulates the ARF expression through the negative feedback mechanisms (Kotake et al., 2011).

## Cellular Senescence and Cardiovascular Aging

As described above, CS is a cell cycle arrest and it presents different characteristics that are categorized as beneficial, neutral, and detrimental based on the conditions (He and Sharpless, 2017). The discovery of senescent cells in the embryos showed that CS also has a role in organogenesis during development. Although these cells express the common biomarker of CS, senescence-associated beta-galactosidase (SA-β-gal), they do not show any DNA damage or senescence-associated secretory phenotype (SASP) cytokines (Muñoz-Espín et al., 2013). Unlike adult senescent cells, they employ p21 (Storer et al., 2013). Furthermore, senescent cells are involved in wound healing, kidney development, placental development, and bone growth (Lozano-Torres et al., 2019). However, within the aging process, the role of senescent cells becomes detrimental and they are involved in age-related diseases such as cardiovascular diseases, cancer, neurodegenerative diseases, pulmonary fibrosis and renal disease. Aging is a biological process that results from the disruption of balance in systems or organisms eventually damaging the homeostasis (Parry and Narita, 2016; Calcinotto et al., 2019). The loss of homeostasis leads to tissue dysfunction causing many age-related diseases (Campisi, 2013). As such, aging organisms experience various chronic diseases and the motivation of research on the biology of aging is to understand the mechanisms behind aging-related pathologies to develop therapies against them. Until now, nine different hallmarks of aging have been determined including the CS (López-Otín et al., 2013), which promotes the idea that aging is affected by multiple parameters instead of one single-cause in the cells (Da Costa et al., 2016). The connection of CS and aging were firstly proved by the studies of INK-ATTAC mouse models in which the removal of p16^INK4A^ cells resulted in reducing the age-related pathologies. These mice studies started a new era for therapeutic approaches to increase the healthspan of organisms. (Baker et al., 2011, 2016).

A recent statistical report released by the American Heart Association (AHA) and National Institutes of Health (NIH) showed that cardiovascular disease (CVD) has a higher incidence than other common diseases, even more than the combination of lung diseases and cancer (Bozaykut et al., 2020a; Virani et al., 2020; Li et al., 2021). Older people are prone to develop any kind of CVD (heart failure, hypertension, stroke, coronary heart disease) and aging has been proposed to be the main risk (Rodgers et al., 2019). Recently, targeting CS, as one of the main hallmarks of aging, has been regarded as a hub for CVD-related aging therapies. It has been shown that people with Human Progeria syndromes, Werner syndrome, and Hutchinson-Gilford progeria syndromes (HGPS), who have a high number of senescent cells even at young ages, develop atherosclerotic plaque burden which accelerates the risk for CVD (Hamczyk, Villa-Bellosta, et al., 2018b). Other studies also have shown that CS is related to arterial diseases including peripheral artery diseases, aortic aneurysm, coronary artery disease, cardiac fibrosis, and heart failure (Cafueri et al., 2012). In addition, heart failure rates increase in the elderly even without any signs of other risk factors such as hypertension, diabetes, obesity or atherosclerosis. The biomechanical and biochemical deterioration of the heart through aging results in left ventricular (LV) dysfunction and arterial stiffening. Diastolic LV dysfunction has also been related to chronic heart failure, which is one of the most observed heart diseases and a considerable proportion of these patients are older people (Benjamin et al., 2019). On the other hand, aortic diseases, such as abdominal aortic aneurysm (AAA) and thoracic aortic aneurysms, are other age-related diseases and it has been shown that there is oxidative DNA damage and telomere attrition in endothelial and vascular smooth muscle cells (VSMCs) of the patients (Cafueri et al., 2012).

### Cardiovascular Cells and Cellular Senescence

During the CVDs, several cell types that are affected by CS are VSMCs, endothelial cells, and macrophages as summarized in Table 1. These cells were shown to have high SA-β-gal levels in people with age-related diseases or in line with old age. Recent studies have also demonstrated the effect of premature CS in atherosclerotic cells (Minamino et al., 2002; Matthews et al., 2006; Hamczyk et al., 2018a). The relationship of the most prominent cardiovascular cells and CS is reviewed below.

**TABLE 1:** Summary of cardiovascular cell senescence pointing out the mechanisms, biomarkers and related diseases.

Cardiac cells	Mechanisms	Biomarkers	Related disease	References
Cardiomyocyte	Telomere damage, Mitochondrial dysfunction, Oxidative stress/ROS	SASP (TGFB2, GDF15, EDN3), p16, p21, SA-β-gal, MMP9, TAF	Cardiac fibrosis, cardiac hypertrophy, heart regeneration, myocardial infarction, cardiomyopathy, arrhythmias	Gude et al., 2018; Childs et al. (2018)
Immune cells	Inflammation, ECM	Telomere shortening, SA-β-gal, TNF-α, IL-6	Myocardial infarction, atherosclerosis, cardiomyocyte hypertrophy	Masso et al. (2015); Ramosa et al., 2017; He et al. (2020); Childs et al. (2018)
VSMCs	Telomere damage, Oxidative stress/ROS, other stressors	Prelamin-A, SA-β-gal, p21, p16, cyclin D1, PDGFRa, TRF2, miRNA-126, HIF1-α	Atherosclerosis, vascular stiffness, AAA, neointima formation, artery calcification, pulmonary hypertension	Alique et al., 2019; Bennett et al. (2016); Matthews et al. (2006); Childs et al. (2018)
Cardiac Stem Cells	INK/ARF pathway, Epigenetic modifications	SA-β-gal, SASP (PAI1, IL-6, IL-8), γH2AX, p16	Chronic heart failure, myocardium, cardiomyopathy	Baker et al. (2016); Lewis and Buffenstein et al. (2016); Cesselli et al., 2018; Cianflone et al. (2020); Childs et al. (2018)
Endothelial Cells	Telomere damage, Mitochondrial dysfunction, Oxidative stress/ROS, Vascular inflammation	SASP (IL-6, VEGF, PAI1, MMP1, MMP3), ICAM-1, TAF, telomere attrition, miRNA-126, HIF1-α	Atherosclerosis, atrial fibrillation, heart failure	Jia et al., 2019; Chen et al. (2018); Childs et al. (2018)

Endothelial cells (ECs) are the building blocks of the inner vascular wall, and they are involved in communication with neighbor cells. These cells are influenced through vascular aging and the number of senescent ECs increases in the arterial walls (Bozaykut, 2019). According to a study of coronary artery diseases, ECs in atherosclerotic plaques are detected with increased SA-β-gal activity (Minamino et al., 2002). The studies on the molecular mechanism of EC senescence reported that in aged people the expression of CS biomarkers (p53, p16^INK4A^, and p21) are increased which can be reversed by exercise. More importantly, EC senescence has been proposed to have an effect on defective vascular endothelial function in aged people (Rossman et al., 2017). In addition, another research showed that endothelial progenitor cells, which are precursors of ECs, are involved in atherosclerosis, and senescence of these cells increases the rate of atherosclerosis in the elderly (Yang et al., 2008). However, the complete mechanisms on how ECs senescence is induced have not been fully understood, which has remained to be investigated.

Vascular Smooth Muscle Cells (VSMCs) are the major cell type that composes the majority of arterial walls. They are critical to maintaining the integrity of blood vessels walls. Besides, VSMCs are involved in the different stages of the atherosclerotic process (Basatemur et al., 2019). In addition, VSMCs play an important role in the development of aortic aneurysm, and fibrotic neointima formation. (Cafueri et al., 2012; Bennett et al., 2016; Komaravolu et al., 2019). Notably, these cells significantly affect atherosclerotic immunity through the artery tertiary lymphoid organs (ATLOs) formation (Hu et al., 2019). Although the role of VSMC senescence in CVDs has been shown in previous studies, there are a few studies on the metabolic regulation of VSMC senescence. Very recently, a study on the sirtuin family demonstrated that SIRT6 protein (but not mRNA) expression is declined in VSMCs in human and mouse atherosclerotic plaques (Grootaert and Bennett, 2021). On the other hand, another study showed that SRT1720 protein has an inhibitory effect on VSMC senescence through the SIRT1 pathway, which was shown by the decrease in SA-β-gal activity and in p21, p53, p16 protein expressions (Sung et al., 2020).

Cardiomyocytes are part of the myocardial tissue affecting their microenvironment with the secretion of pro-inflammatory factors, SASPs, exosomes and 30–40% of the heart consists of cardiomyocytes (Tang et al., 2020). Until recently, cardiomyocytes in adults have been known as post-mitotic cells. Nevertheless, recent studies reported that the proliferative capacities of these cells are protected, yet, their renewal rate is decreasing with aging (Bergman et al., 2009). The CS phenotype of cardiomyocytes is represented by DDR, contractile dysfunction, SASP, mitochondrial defect and ER stress. However, further studies are required to understand the role of senescent cardiomyocytes in cardiac aging and in CVD development (Ock et al., 2016; Tang et al., 2020).

The existence of Cardiac stem cells (CSCs) has been known for almost 20 years in adult mammalian hearts. Progenitor cells have little proportion in tissues, but they hold a potential for differentiating into different cell types in a tissue. CSCs can differentiate into three different cell lineages which are smooth muscles, ECs, and myocytes with their self-renewing, clonogenic and multipotent features (Cianflone et al., 2020). Senescence in these cells is associated with different diseases such as diabetic cardiomyopathy, hypertensive cardiomyopathy, myocarditis and valvular heart diseases (Chimenti et al., 2003).

Immune cells have a role in initiating CVDs through the aging process. The functions of these cells are altered with the CS which is also known as immunosenescence (Rodrigues et al., 2021). Senescent macrophages are one of the pivotal immune cell types for the formation of CVD. They are involved in early atherogenesis and are found to accelerate plaque instability in late phases of the disease (Stoneman et al., 2007). Specifically, leukocyte senescence has been suggested to play a role in initiating plaque formation (Calvert et al., 2011). The link between immunosenescence and CVD is attracting interest and the use of immune cells is proposed as a promising therapeutic approach as discussed later in the review.

## The Tools of Cellular Senescence Research

### Biomarkers for Cellular Senescence

Detection and quantification of the senescent cells (especially *in vivo*) are challenging (Sharpless and Sherr, 2015) and there is no single universal marker to investigate senescent cells. Thus, the combination of several biomarkers is used for the reliable detection of senescence (Di Micco et al., 2021). Although detection of senescent cells is complicated, there are main biomarkers for detecting CS. For instance, physiologically, senescent cells can be distinguished by their flat and extra enlarged morphology. Other than their physiology, there are other biomarkers for the detection of senescent cells *in vivo* and *in vitro*. These biomarkers can be divided into the following four categories: Enzymatic assays, lipofuscin accumulation, proliferative capacity, and molecular biomarkers. On the other hand, the method of choice for the detection of CS also depends on the cell type, physiological context, insult, or the type of stressor (Kirschner et al., 2020).

SA-β-gal activity is a histochemical staining technique used for the detection of accumulated lysosomal enzymes and it is the most widely used biomarker for CS detection. The enzyme β-GAL has a specific working pH range at 6.0 which enables the hydrolysis of X-gal and after the reaction, a deep blue product is released from the cells (Kuilman et al., 2010). Regrettably, due to its presence in pre-senescent, quiescent and immortal cells, it is required to be combined by additional markers such as p21 and p16 expressions (Hall et al., 2017). In addition, senescence-associated heterochromatin foci (SaHF) is another marker for the detection of CS. However, they are mostly found in oncogene-induced senescent cells. Furthermore, modified histone γH2Ax could be used to detect DNA damage from telomere-induced foci (Gire et al., 2014). Another reliable way to analyze the status of DNA replication is through Ki-67, PCNA, or [3H] thymidine.

According to a recent systematic and meta-analysis study that investigates CS variance across the ages and the tissues in humans, even in the same individual, the organs have different levels of CS (Tuttle et al., 2020). Thus, it is vital to choose an appropriate biomarker for effective analysis and the categorization of biomarkers according to study type, such as *in vivo*, *in vitro*, or *ex vivo,* was documented in a recently published review (González-Gualda et al., 2021). Determination of proper markers for tissues in age-related diseases is also related to the type of the disease. The studies on age-related diseases in humans revealed that the most common markers for the detection of CS in heart diseases are p16, SA-β-gal, and p53, followed by SASP, p21, and γH2Ax expressions (Tuttle et al., 2020).

#### Senescence-Associated Secretory Phenotype

In addition to CS biomarkers discussed above, secretory phenotype is being used for the identification of CS (Witkiewicz et al., 2011) since senescent cells secrete chemokines, inflammatory cytokines, growth factors, and matrix metalloproteinases (MMPs) known as SASP. During the aging process, SASP is transcriptionally regulated by CCAAT/enhancer-binding protein β (C/EBP-β) and nuclear factor kappa-B (NF-κB) depending on the CS inducer and context (Acosta et al., 2008; Kuilman et al., 2008; Chien et al., 2011). As a response, interleukin IL-1α (IL-1α) is produced to enhance C/EBPβ and NF-κB activity and amplify SASP signaling through IL-6 and IL-8 production, in the early senescence (Acosta et al., 2008). Moreover, the role of transposable elements which induced cyclic GMP–AMP synthase linked to stimulator of interferon genes (cGAS-STING) pathway was also shown in the activation of SASP (Sedivy et al., 2013; Dou et al., 2017; Li and Chen, 2018).

The communication of senescent cells occurs through this complex series of secretion from senescent cells affecting the nearby cells. Chronic SASP conditions alter the cell environment and induce age-related diseases during the aging process. The components of SASP depend on the context of the disease and might result in distinct outcomes in different cells. Literally, SASPs are the main reason for the double-sided effect of senescent cells as they are known to be beneficial and detrimental (Coppé et al., 2011; Kumari and Jat, 2021). However, during the aging process, their detrimental effect is prominent on neighbour and non-senescent cells and these secretomes are used as the target for senotherapeutic approaches. Recently, senomorphics, a new class of drugs suppressing SASP without eliminating senescent cells have been discovered and will be discussed further.

More recently, these factors have been investigated in the light of an unbiased quantitative analysis and the findings showed that SASP might be investigated under the following four categories: metabolic processes, extracellular matrix/cytoskeleton/cell junctions, regulators of gene expression, and ox-redox factors (Özcan et al., 2016). Different SASP factors could be used for detecting senescent cells in various situations and a recent article created “SASP Atlas” for solving the complexity of SASP components with proteomics study which might facilitate choosing the appropriate biomarkers for senescence according to different types of tissue (Basisty et al., 2020). The role of SASP in inflammaging is suggested previously with different types of inflammatory cytokine secretion. Furthermore, it has been proposed that inflammaging should be considered as a hallmark of CVD (Liu et al., 2020). As summarized in Table 1, different SASP factors are used as biomarkers for CS in different types of cardiac cells.

### Model Organisms to Study Cellular Senescence

**FIGURE 2:**
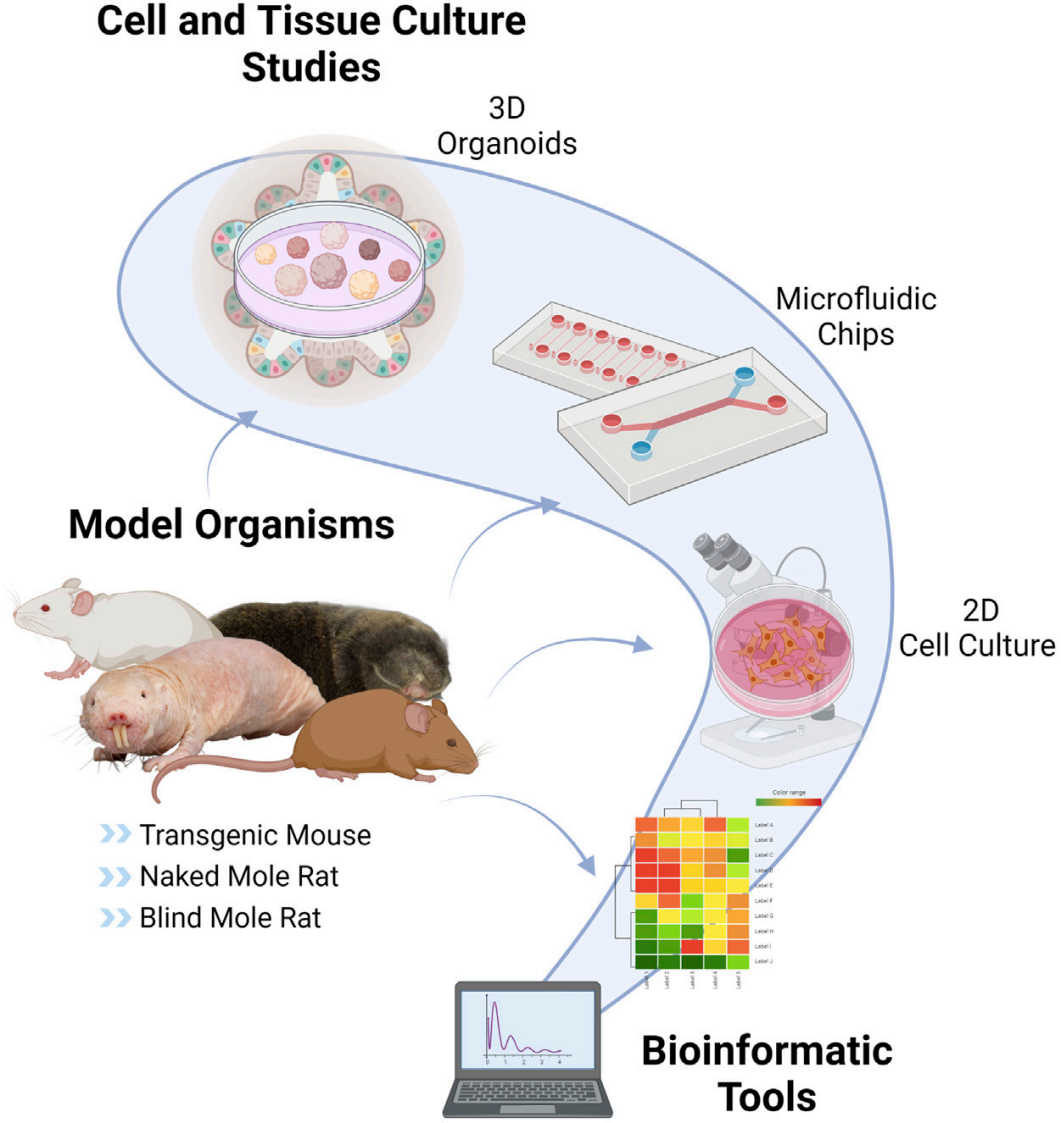
Tools and Models of Cellular Senescence. CS can be studied by using various techniques such as *in vitro* cell cultures, nonhuman models such as usual rodent models (mouse, rat), transgenic mouse models (LMNA progeria mouse, p16-3MR mouse, Burb1 mouse, SAMP/SAMR mouse), special aging models (naked mole-rat, blind mole-rat), 3D organoids, microfluidic chips and bioinformatic applications This figure is created by BioRender.com.

With the increasing popularity of the role of CS on human diseases, the research has expanded to understand the underlying mechanisms by using various experimental models. However, model selection is just as crucial as choosing the proper biomarker. As shown in Figure 2, CS studies can be performed with the help of different types of animal models or organisms, and different experimental and computational approaches.

Nonhuman models are crucial for CS research since it is possible to set up the experimental design to understand the complexity of CS and investigate the potential therapies. However, since main CS research is motivated by the need to resolve the problems mainly in humans, human studies are required for the validation of the therapies besides having ethical considerations. For that reason, 2D cell culture studies have been widely preferred in which the physiological context is not satisfactory. More recently, 3D human organoid cultures have been developed to address these bottlenecks of aging and CS research. In this section, we discuss well-known organisms as well as alternative models and approaches to study CS.

#### SAMP/SAMR Models

The senescence-accelerated mouse (SAM) is established through phenotypic selection to develop a common genetic pool for AKR/J mouse strain. There is a total of 12 SAM strains, including senescence-accelerated mouse resistant (SAMR) and senescence-accelerated mouse prone (SAMP) substrains (Akiguchi et al., 2017), each of which exhibits a specific different phenotype of age-related diseases. Among these, SAMP8, which is characterized by amyloid-beta deposition and abnormal hyperphosphorylation of tau-like neurofibrillary tangles (NFTs) in the hippocampus, a significant decline in learning and memory, cardiac aging, anxiety, and hearing loss, has been widely used in CS research. Although these pathologies make SAMP8 mice suitable for studying Alzheimer’s Disease and other cognitive disorders (Akiguchi et al., 2017; Okouchi et al., 2019; Marie et al., 2018), they are also regarded as a model to study the impacts of aging on cardiovascular health. SAMP8 demonstrates rapid aging, and it has been shown that the main cause of rapid aging of SAMP8 mice is the decreased expression and activity of antioxidant molecules. Furthermore, oxidative stress which is a characterization of SAMP8 mice, is also a risk factor for aging, atherosclerosis, and neurodegenerative diseases, as well (Niedzielska et al., 2016). Since extensive oxidative stress causes irreversible DNA damage, ultimately, it is also associated with CS process.

#### LMNA Progeroid Mouse Models

LMNA progeroid mouse is a transgenic model to study HGPS that was generated by an autosomal recessive mutation in the Lamin A gene. The mutated protein, Lamin A/C (LamA/C), which is an essential component of the nuclear lamina, plays a major role in many nuclear functions such as transcription, cell-cycle progress, DNA repair, DNA replication, and also chromatin organization (Lopez-Soler et al., 2001; Moir and Spann, 2001; Prokocimer et al., 2013). Therefore, mutations of Lamin A protein leads to premature senescence as a consequence of DNA damage, genomic instability, loss of heterochromatin and telomere shortening (Foo et al., 2019). As a result, LMNA mutations cause laminopathies which is the collective group of rare diseases that can be distinguished in systemic or tissue-specific forms and generally show premature aging traits (Camozzi et al., 2014). HGPS, a rare dominant genetic disorder, which is characterized by accelerated atherosclerosis, premature aging, skin and adipose tissue atrophy, and bone resorption (Mounkes and Stewart, 2004), is the most significant form of systemic laminopathies (HGPS, OMIM 176670).

LMNA progeroid mouse reflects HPGS through lamin A, lamin C, and progerin protein expression patterns. In HGPS, vascular calcification is typically observed to lead to calcium-phosphate deposition (CPD) in different layers of the aortic wall, and these progeroid mice are useful to understand the mechanisms of vascular calcification in HPGS. In a study, Osorio et al., define the molecular mechanism of vascular calcification in HPGS by analyzing Lmna^G609G^ knock-in mice. These mice over-express progerin as a result of aberrant splicing which is a consequence of LMNA c.1827C > T (p.G609G) mutation (Osorio et al., 2011). Moreover, it has been reported that there is an increased number of VSMCs and reduced extracellular pyrophosphate expression capacity of VSMCs in progeroid heterozygous Lmna^G609G/+^ mice. In another study, when intracellular ATP levels and extracellular ATP accumulation were analyzed in wild-type and Lmna^G609G/+^ VSMCs, progerin-expressing VSMCs were shown to impair ATP synthesis and mitochondrial function. Finally, inhibition of aortic calcification was observed after 9 weeks of Pyrophosphate treatment in homozygous Lmna^G609G/G609G^ mice (Villa-Bellosta et al., 2013). Collectively, this transgenic model carries major symptoms of HGPS such as skeletal anomalies, impaired somatic growth, shortened lifespan, and cardiovascular alterations, and therefore, is one of the suitable models to study CS-induced aging pathologies.

#### BubR1 Progeroid Mice

BubR1 progeroid mouse is another transgenic mouse model to study the network of CS and aging. The budding uninhibited by benzimidazole related 1 (BubR1) is a crucial protein that plays a key role in mitotic spindle assembly checkpoint. Since its major role is to prevent chromosomes from unequal separation, it helps to maintain chromosomal stability and therefore, BubR1 has direct involvement with CS (Yu, 2002). In addition, alterations in BubR1 expression have been also observed in malignant and premalignant lesions. Studies showed that decreased BubR1 expression causes CS through upregulation of the cell cycle inhibitor p16^INK4A^ (Baker et al., 2004, 2008, 2011; Matsumoto et al., 2007; Kyuragi et al., 2015). However, knockout (BubR1^−/−^) mice did not survive. For that reason, BubR1 mice that have hypomorphic alleles (BubR1^h/h^) and express 10% of normal BubR1 levels were produced for research use (Wijshake et al., 2012).

BubR1 mutant mice exhibit various progeroid (resembling premature aging) phenotypes. In addition, natural aging of wild-type mice is also marked by reduced BubR1 expression, further suggesting that this protein may be a regulator of normal aging process (Baker et al., 2004). Moreover, an increase in senescent hepatocytes and SASP is observed in tissues of BubR1-deficient mice. In a study of mice lacking BubR1 with various senescence-related phenotypes, elevated SASP factors and accumulation of p16^INK4A+^ senescent cells were also observed in adipose and muscle tissues (Baker et al., 2004; Matsumoto et al., 2007; Baker et al., 2008; Baker et al., 2011; Kyuragi et al., 2015).

#### p16-3MR

p16-3MR is a transgenic mouse model that was generated by J. Campisi’s group in 2014. This reporter mouse has three significant features that enable the selective detection, isolation, and depletion of senescent cells (Demaria et al., 2014). Due to the increase of p16^INK4A+^ expression during CS, p16^INK4A+^ has been used as a biomarker to identify senescent cells both *in vitro* and *in vivo* studies. Therefore, this model was generated as a trimodal reporter combined with the p16^INK4A+^ gene. Under the control of the p16^INK4A+^ promoter, it contains functional domains of a synthetic Renilla luciferase (LUC) to permit the detection of 3MR-expressing cells by luminescence both *in vitro* and *in vivo*; monomeric red fluorescent protein (mRFP) to permit the isolation of senescent cells from tissues, and lastly; truncated herpes simplex virus 1 (HSV-1) thymidine kinase (HSV-TK) to permit selective elimination of senescent cells (Demaria et al., 2014). This elimination occurs by the combinational use of HSV-TK with ganciclovir (GVC) that acts as a toxic DNA chain terminator in senescent cells, breaking down the mitochondrial DNA and, hence, causing apoptosis (Laberge et al., 2013).

The clearance of senescent cells has been shown to delay or reduce aging phenotypes through p16-3MR model since it has a mechanism that selectively and efficiently targets p16^INK4A+^ senescent cells such as endothelial-like cells, foamy VSMC-like cells, and foamy macrophage-like cells by GCV administration. p16^INK4A+^ senescent cells are known to be uniformly deleterious throughout atherogenesis. It has been shown that the elimination of these cells either via pharmacologic or transgenic approaches inhibits disease-related detrimental pathologies such as fibrous cap thinning and elastic fiber degeneration and reverses atherosclerosis (Childs et al., 2016). Collectively, these studies have shown that p16-3MR is an excellent model to study the inducible depletion of the p16^INK4A+^ senescent cells, and therefore, CS-related diseases such as CVDs.

### Special Aging Models: Naked Mole Rat and Blind Mole Rat

Most of the rodents are known to have a short lifespan, however, there are superior species that evolved longer lifespans. Among these, naked mole-rat (NMR) and blind mole rat (BMR) are the most notable subterranean species and their extraordinary nature makes them important model organisms for the biology of aging research. One of the main differences between NMR and BMR is their social hierarchy. NMRs are eusocial animals living in communities with one queen that reproduce the rest of the community whereas BMRs are solitary and aggressive. NMRs and BMRs maximum lifespans are 32 and 21 years, respectively. In addition their longer lifespan, they have also evolved anti-cancer resistance, hypoxia tolerance, and many adaptation strategies for extreme conditions, as well. Therefore, understanding the mechanisms underlying the extreme adaptations of these special rodents may contribute to novel therapies for human healthspan (Azpurua and Seluanov, 2012).

#### Naked Mole Rat

NMR is a eusocial species endemic to the deserts of East Africa, with an extraordinary lifespan of more than 30 years (Edrey et al., 2011; Lewis and Buffenstein, 2016; Bozaykut, 2021). NMR lifespan studies based on more than three thousand data by Kaplan-Meier analyzes showed no significant increase in age-related mortality when compared to other mammals (Kirkwood, 2015) pointing out NMR as an ageless organism (Ruby et al., 2018).

In NMR, cellular senescence is evolved as a defense mechanism against tumor formation by restricting the proliferation of damaged or premalignant cells. Also, it has been linked to the aging process and age-related pathologies. On the other hand, while being longest-lived rodent, NMR has resistance to various age-related diseases (Tacutu et al., 2011; Yanai and Fraifeld, 2018). Interestingly, NMRs have been shown to undergo the following three different types of CS that are DNA damage-induced, oncogene-induced, and developmental CS (Zhao et al., 2021) although they do not show age-related phenotypes until the very late stages of their lives (Buffenstein, 2008). In addition, aged NMRs also do not show deterioration in cardiovascular function, muscle structure or function, bone quality, cognitive functions, or any age-related pathologies (Pinto et al., 2010; Edrey et al., 2011; Grimes et al., 2014; Stoll et al., 2016). Surprisingly, in a study that compared young and old individuals, higher cytosolic and mitochondrial ROS were found even in younger individuals (Labinskyy et al., 2006). In contrast, older individuals resist oxidative stress (Andziak and Buffenstein, 2006) and the lack of oxidative stress activation during aging avoids the perturbation of mitochondrial function, and NMRs escape from growth arrest.

It has been reported that the main reason behind cancer resistance is early contact inhibition (ECI), which is considered as the main barrier to prevent the overgrowth of the cells (Seluanov et al., 2009). Furthermore, while hypoxia tolerance is very low in the majority of mammals (Dzal et al., 2015), NMRs can survive even in extreme hypoxic and anoxic conditions. Relatively, NMR’s have been shown to develop special metabolic adaptations under anoxic conditions in which they use fructose as a fuel of glycolytic metabolism (Park et al., 2017).

In addition to metabolic and epigenetic adaptations in NMR, Faulkes et al. defined the cardiac metabolic profile and biochemical alterations. In addition, according to the previous metabolic profiling reports (Park et al., 2017), NMRs were shown to have an increased glycolysis, lactate, and glutathione rate which suggests the increased resistance to oxidative stress. Furthermore, succinate/fumarate ratio was also demonsrated (Faulkes et al., 2019). Therefore, a variety of metabolic adaptations observed in the NMR heart could be responsible to increase the survival ability and protecting NMRs from age-related CVD.

It has been observed that there is a high level of lipid peroxidation in the heart tissue of NMRs thus, it can prevent the progression to fatal cardiac disease (Grimes et al., 2012). In addition to this feature, the conserved cardiovascular function (Grimes et al., 2014) which is not deteriorated by age makes this organism a perfect candidate to study CS and age-related cardiovascular pathologies.

#### Blind Mole Rat

Similar to NMR, BMR is an underground rodent that is highly resistant to hypoxia and cancer, with a reported maximum lifespan of 21 years (Avivi et al., 2005; Shams et al., 2005; Edrey et al., 2012; Gorbunova et al., 2012; Schmidt et al., 2017). Furthermore, while tumor formation can easily be induced in mice and rats, BMRs are remarkably resistant to exogenous carcinogens, and in 40 years of observation, not a single incidence of spontaneous cancer was observed (Gorbunova et al., 2012; Manov et al., 2013). Several studies on the superior cancer resistance of BMRs demonstrated that BMR cells exhibit uniform, vigorous proliferation and are able to achieve high-density assembly without ECI. Instead of ECI, BMR cells pass through a relatively small number of population doublings while proinflammatory IFN-β is released as a response that is driven by p53/RB pathway. Followingly, cells die because of the massive necrosis within 3 days, named as concerted cell death (Gorbunova et al., 2012).

Moreover, a recent study showed that replicative senescence in BMR fibroblasts was manifested by the increased activity of SA-β-gal and overexpression of p16, p21, and p53 mRNA expressions (Odeh et al., 2020). Suprisingly, in senescent BMR fibroblasts, SASP, as one of the important features of CS, was found to be undetectable or decreased unlike senescent human and mouse fibroblasts (Odeh et al., 2020). The study proposed that, the DNA damage required for the activation of SASP in BMR senescent cells was not sufficient, connecting with the data on the increased DNA repair capacity of BMRs (Domankevich et al., 2018). The same study also suggested that the increased p65 phosphorylation was also not enough to activate NF-κB pathway which is accepted as one of the main SASP regulator (Odeh et al., 2020). In addition, a very recent study interestingly reported the activation of retrotransposable elements (RTEs) that in turn induces cGAS–STING pathway. As cGAS–STING pathway is connected to SASP as explained above, the role of cGAS-STING pathway on SASP status of BMR during aging remains as an important question to be resolved (Zhao et al., 2018).

Although these data suggest that NMR and BMR, two special aging models, are likely to evolve different adaptations during the aging process, further investigations in order to understand these adaptations are needed to develop alternative ways to treat CS-related pathologies.

### Cell and Tissue Culture Studies

Since the motivation to study the biology of aging is the need to resolve human age-related pathologies, human models are emerging. By using *in vitro* 2D cell culture methods, it is possible to induce CS and therefore, observe CS progress in various cell types such as human primary dermal fibroblasts (Galvis et al., 2019), human fetal fibroblasts (Hayflick and Moorhead, 1961), human primary bone marrow mesenchymal stromal cells (BM-MSCs) (Antonioli et al., 2019) and IMR90 (Xu et al., 2020). However, due to its highly heterogeneous phenotype and complexity of human aging, 2D cell cultures can not fully meet the biology of human aging. Model systems demonstrating the features of environmental interactions, stochastic and genetic-epigenetic variables such 3D organoids or microfluid chips have been recently developed to solve these challenges of human aging studies as described below (Cevenini et al., 2008).

#### 3D Organoid Cultures

Specifically, studying human CS is challenging as the number of senescent cells in an organism is very few to observe, in addition to the long aging process of humans. These challenges lead researchers to study senescent cells *in vitro* (González-Gualda et al., 2021) however, as discussed above, 2D cell cultures do not reflect the true representation of human aging. Very recently, researchers have been focused on more advanced methods to study senescence and one of the most promising techniques to study CS is proposed as 3D organoid cultures (Torrens-Mas et al., 2021). Organoid systems are simplified organs developed to model many human tissues and diseases (Hu et al., 2018), reconstructing physiological 3D tissue structure and cellular composition *in vitro* (Simian and Bissell, 2017). Organoids can be derived from primary cells and tissues or from sources such as pluripotent stem cells (PSCs) (Barkauskas et al., 2017). On the other hand, a recent study suggested intestinal epithelial organoids are appropriate for aging studies as shown by the SA-β-gal accumulation, the decrease in DNA methyltransferase expression, and the increase in p21 expression in organoids of aged mice (Uchida et al., 2019).

The use of organoids in aging studies was shown in the experimental setup of intestinal organoids from young and old samples. Epigenetic changes resulting in stem cell dysfunction and the reduction in Wnt signaling explain the reduced organoid generation efficiency for aged mice and humans when compared to younger individuals (Mihaylova et al., 2018; Pentinmikko et al., 2019; Uchida et al., 2019; He et al., 2020). In addition, aged organoids showed decreased DNA methyltransferases as well as increased CS markers including SA-β-gal and p21 and p16 expressions (Christoph et al., 2021). Furthermore, one of the well-described longevity strategies is the dietary intervention has been also validated in a recent study. The study demonstrated that 24-h fasting resulted in enhanced organoid formation and self-renewal potential in aged mice (Mihaylova et al., 2018). In addition, improved organoid formation efficiency has been also demonstrated in calorie-restricted mice that are explained by the alternations in mTORC and SIRT1 signaling both of which are the essential pathways in aging (Yilmaz et al., 2012; Igarashi and Guarente., 2016; Igarashi et al., 2019).

Skin aging studies mainly rely on the *in vitro* cell cultures in which CS can be induced or more recently on the skin equivalents of 3D culture of fibroblasts and keratinocytes isolated from aged donors. These 3D models have been shown to successfully represent many features of skin aging (Diekmann et al., 2016). As such, p16 levels were shown to vary significantly in young and old-derived skin equivalents and p16 was suggested to drive other changes related to skin aging (Adamus et al., 2014). Furthermore, skin equivalents were also used to test the effect of fat-derived stem cells on CS and it was shown to delay the expression of senescence markers (Odile Damour, 2014).

Another 3D organoid model has been developed by cortical neurons that are differentiated from human iPSC were shown to represent typical features of senescent cells, such as increased SA-β-gal activity, p16/p21 expressions, and inflammatory cytokines (Shaker et al., 2021). This senescent phenotype was also accompanied by the significant downregulation of Klotho, a type I transmembrane protein with anti-aging properties (Kuro-o., 2011; Massó et al., 2015; Shaker et al., 2021).

Since organoids have the ability to histologically recapitulate human tissues *in vivo*, this technology holds the potential to test potential longevity drugs that target the hallmarks of aging including CS, while paving the way to personalized interventions. However, organoid cultures also lack some physiological features of the organisms such as vascularization and further developments are needed to better represent the physiology of human aging (Torrens-Mas et al., 2021).

### Microfluidic Chips

Since 2D culture studies are restricted due to the lack of environmental factors, microfluidics is yet another emerging technology for the manipulation of the environment at the microscale level. These systems have the ability to copy the environment of organs, vessels, or tissues through various regulations (Wu et al., 2020). This novel technique has been recently initiated to study senescent cells and 3D filter senescence chips were developed to isolate and to remove senescent cells from biofluidics which enables low cell damage while filtrating senescent cells (Chen et al., 2018). In a recent study, an *in vitro* 3D model which is called “dermis-on-a-chip” that mimics the blood vessels with skin fibroblasts was developed. This vessel is embedded in the chip along with collagen-type-1 and senescent fibroblasts were applied to the chip for the observation of CS effect in the microchip environment. This study provided evidence on how to optimize senescent cell concentration in the experimental setup while enabling observing the role of CS (Pauty et al., 2021).

Another microfluidic study displayed a tube-free microfluidic platform for VSMC culture where extracellular matrix coating, VSMC seeding, culture, and immunostaining can be performed (Wei et al., 2014). In this approach, VSMCs are seeded into microfluidic devices by even distribution. In comparison to bare glass surfaces, VSMC proliferation and phenotype variations caused by extracellular matrix coated substrates were explored in time sequence. Furthermore, developed a progeria-on-a-chip model to study HGPS which is driven by vascular aging. This biomimetic microfluidic model was designed with VSMCs and the authors showed the effect of biomechanical strain by comparing the SMCs from healthy and HGPS donors. The model revealed a new strained-derived mechanism with *in vitro* studies (Ribas et al., 2017). Overall, newly enhanced techniques on CS-related studies have had contributions to diagnostics and drug applications on CVD and other age-related pathologies.

### Bioinformatic Approaches

After the discovery of senescent cells, scientists have put an enormous effort to identify universal markers for the characterization of the CS state. One of the reasons that make it difficult to identify such markers, is the high heterogeneity and complexity of the senescence phenotype. Because of this complex feature of CS, detecting senescence is only possible by considering the combination of multiple biomarkers within the same sample (Sharpless and Sherr, 2015). However, during the identification of CS and testing the potential treatments, it is important to have not only highly sensitive biomarkers but also cost-effective techniques are needed. Since bioinformatic studies provide a significant reduction in cost and time, it has become an important tool of science, making it possible to observe the development of senescence at the single-cell level (Wiley et al., 2017). With the help of computational approaches and high-throughput techniques such as proteomics, genomics, transcriptomics, metabolomics, it is possible to observe the development of CS through common biomarkers and SASP phenotypes, and to design therapeutic targets for senotherapeuti̇c approaches accordingly (Hernandez-Segura et al., 2018).

On the other hand, with the help of omics technologies, it is also possible to propose new drug candidates such as by repurposing drugs that already exist. Besides the discovery of novel senotherapeutics, drug repurposing provides faster progress, fewer cost, and low attrition rates than the traditional drug discoveries (Pushpakom et al., 2019). Computational technologies offer a great number of platforms such as databases, tools, and servers to identify candidates of repurposing drugs by fishing genes and specific targets while investigating the presence of the interactions with a target of known drugs (Nabirotchkin et al., 2020).

## Strategies Targeting Cellular Senescence: Senotherapeutics

Senescent cell accumulation causes various consequences leading to age-related pathologies. Ultimately, prevention of CS or clearance of senescent cells may be a promising therapeutic approach for age-related diseases. Senotherapeutics is the common name given to the class of drugs that target senescence-associated phenotypes and/or senescent cells to prevent age-related diseases. Senotherapeutics can be classified into two diverse groups named senolytics and senomorphics each of which modulates CS in different ways (Kim and Kim, 2019). Senomorphics mainly block the senescence-associated phenotypes to prevent/delay CS state without affecting the total number of senescent cells. On the other hand, senolytics aim to selectively kill senescent cells by inducing cell death and reducing the total number of senescent cells (Si and Liu, 2014; Kim and Kim, 2019). Besides senomorphics and senolytics, preventing senescence formation before they accumulate and using immune cells for the removal of senescent cells have also been proposed as non-pharmacological therapeutic approaches (Amaya-Montoya et al., 2020).

### Senolytics

The most common feature of senescent cells is their resistance to apoptosis and they avoid cell death through the expression of prosurvival proteins. The discovery of the senolytic drugs was accomplished by showing that inhibitors of these prosurvival proteins, such as redesigned cancer chemotherapeutics, can also kill senescent cells (Guerrero et al., 2019). Until now, several classes of senolytics have been discovered that include Bcl-2 family inhibitors, p53 binding inhibitors, kinase inhibitors, heat shock protein 90 inhibitors, histone deacetylase inhibitors and natural compounds (Niedernhofer and Robbins, 2018).

The first senolytic drugs were designed on the hypothesis that senescent cells are more susceptible to the inhibition of survival networks than their normal counterparts. This hypothesis led to the discovery of dasatinib (D), a multityrosine kinase inhibitor, and quercetin (Q), a flavonol, that improved cardiac function (Zhu et al., 2015). Regardingly, it was shown that the combination of D + Q resulted in the removal of senescent cells while reducing vasomotor dysfunction in old mice in addition to decreasing aortic calcification (Roos et al., 2016). According to another study, D + Q combination decreased the senescent cell burden in aged Ercc1−/Δ-progeroid mice which was accompanied by the healthspan extension and the reduction of age-related pathologies (Ogrodnik et al., 2017). This combinational therapy also reduced senescent cell load in aged, irradiated, and progeroid mice, while improving cardiovascular and physical function (Xu et al., 2018).

B-cell lymphoma 2 (BCL-2) family inhibitors have been demonstrated to have the capacity to remove senescent cells by blocking prosurvival pathways as such, ABT737 inhibits the interaction of antiapoptotic proteins with proapoptotic proteins which consequently leads the senescent cells to apoptosis (Ovadya and Krizhanovsky 2018). Navitoclax (ABT263), which is the orally bioavailable analog of ABT737, is another frequently studied potential senolytic that has been shown to improve a wide range of CS pathologies *in vivo* (Van Deursen., 2019). Navitoclax appeared to be effective against human umbilical vein epithelial cells (HUVECs) and IMR90 human lung fibroblasts, although it was not effective for primary preadipocytes. Navitoclax targets BCL-2, BCL-xL, MCL-1, and its activity is closely related to the differential expression levels of these targets in various types of senescent cells (Kang, 2019). In addition, Navitoclax removes the senescent cells during atherogenesis and, therefore, has the potential to address age-related CVD pathologies by the prevention of newly developing lesions (Childs et al., 2018).

p53 is another key component of CS and p53/p21 axis has the capacity for the development of novel senolytics. As such, a peptide that inhibits the interaction of p53 with FOXO4, was shown to release p53 and ultimately, induce apoptosis of senescent cells in old mice (Baar et al., 2017). HSP90 inhibitors are yet defined as another class of senolytics. Studies on Ercc1 −/∆ mice, a mouse model of human progeroid syndrome showed that the treatment of HSP90 inhibitor, 17-DMAG, prolonged the healthspan of these mice by delaying the onset of several age-related symptoms and reducing p16^INK4A^ expression (Fuhrmann-Stroissnigg et al., 2017).

First-in-human clinical trials with senolytic drugs were also published in 2019 with patients diagnosed with idiopathic pulmonary fibrosis (IPF) which is typically characterized by CS and a shorter lifespan. The results of the clinical trials showed that the physical performance of the patients was increased after 3 weeks of treatment with (D + Q) (Justice et al., 2019; Ellison-Hughes, 2020) pointing the significant potential of senolytics to target age-related CVDs.

### Senomorphics

Another strategy to therapeutically target senescent cells is to reduce their disease-causing phenotype, termed as senomorphics (Fuhrmann-Stroissnigg et al., 2017), also known as senostatics (Kang, 2019). The principle of senomorphics is to alter their ability to maintain a stable growth arrest or to disrupt the essential features of senescence, mainly SASP production and secretion while keeping cells alive. This approach has the capacity to interfere with the proinflammatory nature of senescent cells and potentially avoid the main pathologies of aging and aging-related disease (Herranz et al., 2015).

Senomorphics are suggested to modulate the senescent cells, senescence-associated phenotypes, and senescence-related signal pathways without inducing apoptosis of senescent cells. Telomerase activators (Liu et al., 2017), mTOR inhibitors (Lamming et al., 2013), sirtuin activators (Hubbard and Sinclair, 2014), antioxidants (Si and Liu, 2014), anti-inflammatory agents that target senoinflammation or inflammaging (Soto-Gamez and Demaria, 2017), proteasome activators (Kim and Kim, 2019), and autophagy activators (Nakamura and Yoshimori, 2018) have been proposed as candidate senomorphics. Furthermore, simvastatin which is a drug that belongs to the class of statins and it has been mainly used to prevent increased cholesterol levels (Taylor et al., 2013). However, simvastatin was also shown to intercept SASP in senescent fibroblasts and cell cycle growth arrest in endothelial progenitor cells (Assmus et al., 2003; Liu et al., 2015).

### Immune Surveillance

Immune therapy is yet another approach to clear senescent cells, by increasing their capacity for targeting senescent cells. Different immune cell-based therapeutic approaches are used to treat diseases and these approaches might be enhanced to target senescent cells (Moutsatsou et al., 2019). As such, chimeric antigen receptor (CAR) T cell therapy is a novel approach that has been used for treating diseases such as cancer. These cells are genetically modified with synthetic antigens to target specific cells with reconstruction (Yu et al., 2017). Very recently, a cell-surface protein which is the urokinase-type plasminogen activator receptor (uPAR) was observed in senescent cells with high induction. This revolutionary study also showed that CART cells, that specifically were designed to target uPAR proteins, eliminate the senescent cells both *in vitro* and *in vivo* studies (Amor et al., 2020).

Moreover, natural killer (NK) cells have the capability to target senescent cells and the modification of NK cells, as in CART cells, is a recent technique (Xie et al., 2020). Other immune cells have also various roles in CS and these cells also have the potential to develop novel senotherapeutic approaches as explained in detail by Prata et al. (Prata et al., 2018). Currently, different types of immune therapies are being developed, and converting these immune cells into senotherapeutics is a new and promising research area that may pave the way to cure CS-related diseases.

### Pre-Senescence Therapy: Prevention of Senescence

Another different therapeutic approach for CS-related diseases is to prevent senescence accumulation before cells enter CS state while extending the lifespan of cells. Several studies showed that caloric restriction has a positive effect on lifespan in various models (Mendelsohn and Larrick, 2018). More particularly, calorie-restricted mice were shown to have lower telomere-associated DNA damage foci (TAF) in hepatocyte cells (Ogrodnik et al., 2017). Caloric restriction also elevated β-HB, a type of ketone body, which resulted in the reduced aging-related neurodegeneration (Paoli et al., 2014) and a recent study also reported that inducing cellular quiescence with β-HB has an inhibitory effect on replicative senescence and stress-induced premature senescence (Han et al., 2018). Another research investigated the effect of exercise and diet on senescent cells and showed that fast-food diet led to the increased expression of senescence markers (SASP, SA-β-gal, p16 and EGFP) in the adipose tissue of mice. Interestingly, expression of SASP factors was inhibited after fast-food fed mice performed exercise (Schafer et al., 2016). Taken together, these findings highlight new approaches and potential treatments for CS-related diseases which is to prevent the senescence accumulation before they enter senescence. Indeed, the possibility of senotherapies becoming clinical drugs in real life might have challenges in efficacy and for a maximised effect, treatments should be considered either in a proactive manner or in the very early stages.

## Clinical Perspectives and Conclusion

Even though several molecular mechanisms of CS have been elucidated, other possible pathways may contribute to the development of CS during aging. Although there have been many studies focused on CS biology and its mechanisms of action, the lack of a universal marker for the detection of CS is still an important challenge due to the complexity of the process. Therefore, future studies are expected to lead to the discovery of novel pathways pointing out particular biomarkers based on the diseases or tissue types, instead of focusing on one universal marker.

Most of the resources, now, also have shown that not all the model systems are exactly suitable to study CS in various conditions, disease states and cell/tissue types. For that reason, it is suggested that, instead of choosing one single model, the combination of the results from various models would provide a better understanding of this complex phenomena (Barros et al., 2021). While there are several studies on alternative aging models such as NMR and BMR, there is no sufficient data to fully understand how these unique species adapt CS mechanisms to achieve healthy aging. It should be noted that new perspectives are required to go beyond the current CS knowledge and further studies with long-lived species may pave the way for humans to switch to their longevity status.

For decades, CS studies have been conducted by *in vitro* cell culture systems or nonhuman models. However, within the current scientific innovations, now, researchers are focusing on more advanced and reliable techniques for humans. Of them, 3D patient-derived organoid studies have recently gained interest in aging and age-related diseases by their potential to change the dependence on non-human model systems (Uchida et al., 2019). This technique might solve two of the major bottlenecks of aging, which are the discovery of new biomarkers of human aging and the translation of preclinical studies to humans. Furthermore, recent rapid progress in computational techniques will aid to improve the efficiency of the senotherapeutic approaches (Regnault et al., 2021) that would fit better to human pathologies and will enhance the possibility of successful human clinicals.

Lastly, senotherapeutic approaches have been used in the treatment of various age-related diseases including CVD in the last couple of years (Anderson et al., 2019; Walaszczyk et al., 2019) and new methods are in the process (Balistreri et al., 2021). Although many mechanism-based approaches for targeting senescent cells have been validated, their side effects are likely to limit therapeutic use. Besides, the long effect of killing senescent cells in humans stands a big question mark. That’s why, interest in different therapeutic methods, including immune surveillance and senomorphics has been raised. Successful translation of preclinical studies with the help of alternative models and techniques is emerging for the novel therapeutic approaches and may facilitate the way to the extension of healthspan and lifespan in humans.
